# Design of Healthy Snack Based on Kiwifruit

**DOI:** 10.3390/molecules25143309

**Published:** 2020-07-21

**Authors:** Urszula Tylewicz, Malgorzata Nowacka, Katarzyna Rybak, Kinga Drozdzal, Marco Dalla Rosa, Massimo Mozzon

**Affiliations:** 1 Department of Agricultural and Food Sciences, University of Bologna, Piazza Goidanich 60, 47521 Cesena, Italy; urszula.tylewicz@unibo.it (U.T.); marco.dalla.rosa@unibo.it (M.D.R.); 2 Interdepartmental Centre for Agri-Food Industrial Research, University of Bologna, Via Quinto Bucci 336, 47521 Cesena, Italy; 3 Department of Food Engineering and Process Management, Institute of Food Sciences, Warsaw University of Life Sciences–SGGW, 02-787 Warsaw, Poland; katarzyna_rybak@sggw.edu.pl (K.R.); kingadro9@gmail.com (K.D.); 4 Department of Agricultural, Food and Environmental Sciences, Università Politecnica delle Marche, Via Brecce Bianche 10, 60131 Ancona, Italy; m.mozzon@staff.univpm.it

**Keywords:** snack, kiwifruit, bioactive compounds, polyphenols, flavonoids, vitamin C, antioxidant activity

## Abstract

Kiwifruit is an excellent source of vitamin C and other bioactive compounds, which contribute to its high antioxidant activity. However, the fruits with small size and low weight are considered waste and are unprofitable; therefore, the production of healthy kiwifruit-based dried snacks, which contain a lot of health-beneficial ingredients, could be a viable alternative for their use. The aim of this study was to develop formulations and methods to produce attractive and nutritionally valuable dried snacks based on yellow kiwifruit. Three different puree formulations (kiwifruit; fennel; and strawberry, lemon, or spinach) with or without addition of sugar were subjected to two drying methods: freeze-drying (fruit bars) and conventional hot air drying (fruit leathers). The obtained products were analysed for their content of total polyphenols (TPs), flavonoids, and vitamin C, as well as their antioxidant activity. The results showed that snacks prepared by freeze-drying (fruit bars) presented higher TP, vitamin C, and flavonoids content than those prepared by convective drying; however, the antioxidant activity did not always follow this trend. The amount of bioactive compounds depended on the formulation used for the preparation of snacks. The effect of the sugar addition seems to be strictly related to the mix used and specific bioactive compound investigated.

## 1. Introduction

Recently, consumers are more interested in sustainable food production, owing to the growing awareness of environmental pollution and the high amount of waste generated during conventional food processing. Moreover, they are searching for food products with improved safety, as well as healthy aspects [1,2].

The trends of healthiness and sustainability have triggered consumers into choosing the products as natural as possible with a clean label. The clean label issue appeared in the 1980s and has been growing continuously [3]. There is no a widely accepted definition of a clean label, however, studies conducted by Moskowitz et al. [4] showed that consumers try to avoid food products with unfamiliar additives/ingredients, which are often associated with artificial chemicals and products not obtained in a natural way (e.g., genetically modified organisms—GMOs). Moreover, changes in consumer habits and lack of time to prepare a proper meal at home drive the consumers to eat outside of home, often choosing snacks [5]. Hence the food industry’s focus on developing healthy fruit snacks, which could provide consumers with nutrient-dense energy snacks. Healthy snacks are defined as snacks containing a high number of beneficial ingredients that have a positive effect on human health, and usually well-balanced nutrients [2]. Dried fruits are considered a healthier alternative to popular snacks rich in sugar or salt and the recommendations for their consumption are constantly present in the dietary guidelines of many countries [6]. As a result of their production process, a concentration of many naturally occurring compounds (especially in terms of energy, minerals, antioxidants, and fiber) is obtained, which is associated with their nutritional values and exceptional sensory attractiveness [7].

Different drying methods could be used to obtain functional fruit snacks, which allow to increase the shelf-life and facilitate the handling of the products. On the basis of the drying methods used, different products could be obtained. Convective drying of a mixture of fruit puree or concentrated fruit juice with other ingredients such as sugars, hydrocolloids, and so on is used to obtain fruit leathers, a thin flexible layer that shows a texture similar to a soft leather [2,8]. However, owing to a long exposure of the products to high temperatures, thermolabile compounds could be lost and some other chemical and physical changes may occur in the products [9]. The freeze-drying process, using lower temperatures and reduced pressure, is more expensive, but allows a much better retention of bioactive ingredients [6]. This method allows to produce fruit bars using fruit puree mixed with hydrocolloids [10]. Both snacks, fruit leathers and bars, are becoming a popular food format, mainly because of the growing consumer demand for healthy, natural, and convenient foods [11].

An advantage of the manufacturing of these kinds of products is that the waste arising from the fruit and vegetables processing or fruits with lesser value in terms of size and shape could be used to prepare the puree mix. Concerning kiwifruit, those with the weight lower than 65 g are considered waste and are unprofitable, as they are used in the production of fruit juices or in the energy supply chain [12]. Therefore, their use in the production of fruit snacks could be a good alternative for waste management and to increase their added value.

Kiwifruit is a good source of different bioactive compounds and thus shows high antioxidant activity. Kiwifruit is a good source of vitamin C [13,14] and polyphenols [15,16]. Flavonoids are the most abundant phenolic substances, showing a range from 81.50 to 161.38 mg catechin equivalents (CTE)/100 g fresh weight (FW), and about 120 mg CTE/100 g in Jintao cultivar [14].

Therefore, the aim of this study was to develop formulations (Table 1) based on wasted yellow kiwifruit (cultivar Jintao) with the addition of other fruit and vegetables and pectin as a structuring agent to produce dried snacks. Two drying methods were studied, convective drying and freeze-drying, to obtain fruit leathers and bars, respectively. The products were analyzed for their content of bioactive compounds (vitamin C, total polyphenols, and flavonoids) and their antioxidant activity using the ABTS assay (assay with 2,2-azino-bis(3-ethylbenzothiazoline-6-sulfonate)).

## 2. Results

### 2.1. Vitamin C Content

**Figure 1:**
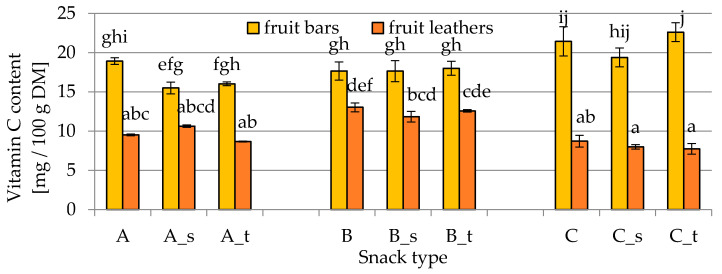
Vitamin C content in fruit bars and leathers (A, B, C—different composition; s—sucrose addition; t—trehalose addition; DM—dry matter) obtained using liquid chromatography, as described in Section 4.2.1. Different letters above the columns indicate significant differences (*α* = 0.05) between all considered samples.

The vitamin C content of fruit leathers and bars is shown in Figure 1. The vitamin C content was influenced by both the composition of fruit snacks and the drying method. The highest content of vitamin C (about 22 mg/100 g dry matter (DM)) was observed in fruit bars prepared with the highest kiwifruit content and with the addition of fennel and spinach, while the lowest (about 8 mg/100 g DM) was observed in fruit leathers prepared with the same formulation. In fact, the method of drying and the associated temperature significantly influenced this parameter. Independently of the formulation used, the fruit bars obtained by freeze-drying were characterized by much higher retention of vitamin C than fruit leather dried with hot air. The results clearly showed that the higher temperature together with an oxygen-rich environment during air drying significantly reduced the ascorbic acid content. The addition of sugars did not influence (*p* > 0.05) the vitamin C content, regardless of whether sucrose or trehalose was used (Figure 1).

### 2.2. Total Polyphenol Content (TPC)

**Figure 2:**
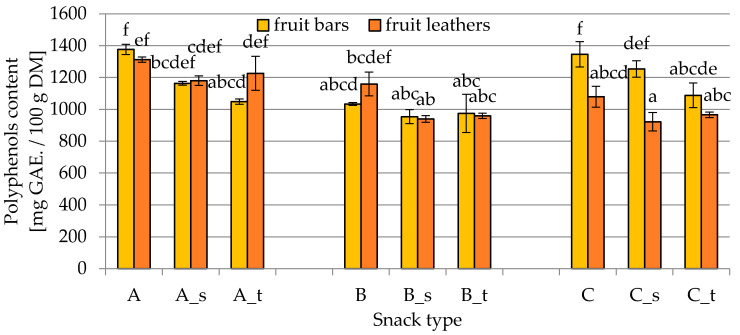
Polyphenols content in fruit bars and leathers (A, B, C—different composition; s—sucrose addition; t—trehalose addition) determined by the Folin–Ciocalteu method, as described in Section 4.2.2. Different letters above the columns indicate significant differences (*α* = 0.05) between all considered samples.

The total polyphenol content (TPC) in fruit leathers and bars is shown in Figure 2. In general, lower values of this parameter were obtained in products prepared with the addition of fennel and lemon juice and peel (formulation B). Drying methods in most of the cases did not significantly influence the TPC among snacks of the same composition. The exception was the C snack, where the fruit bars were characterized by a higher content in polyphenols compared with fruit leathers, showing values of 1346 ± 79 and 1079 ± 66 mg gallic acid GAE/100 g DM, respectively, in the control C snack, and 1254 ± 51 and 922 ± 58 mg GAE/100 g DM, respectively, in the snack with the addition of sucrose (C_s).

### 2.3. Flavonoid Content

**Figure 3:**
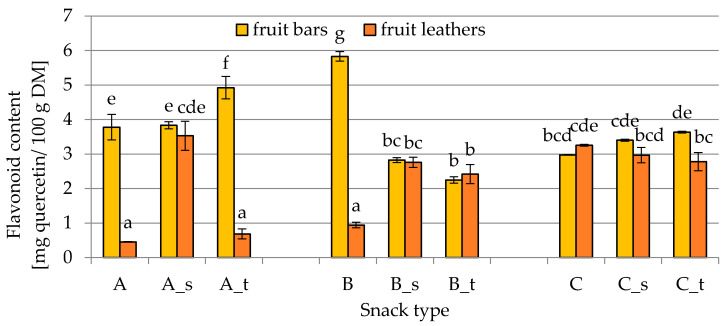
Flavonoid content in fruit bars and leathers (A, B, C—different composition; s—sucrose addition; t—trehalose addition) determined spectrophotometrically, as described in Section 4.2.3. Different letters above the columns indicate significant differences (*α* = 0.05) between all considered samples.

The flavonoid content in fruit leathers and bars is shown in Figure 3. The behavior of flavonoid content in both snacks strongly depended on the formulation used, showing the highest value (5.83 ± 0.14 mg quercetin/100 g DM) in fruit bars B (with fennel, strawberries, and lemon juice) without the addition of sugars, followed by fruit bars A with the addition of trehalose (4.92 ± 0.33 mg quercetin/100 g DM) and fruit bars A without sugar addition (3.78 ± 0.37 mg quercetin/100 g DM). The fruit leathers prepared with the same formulations presented a significantly lower flavonoid content, showing values of 0.94 ± 0.08, 0.68 ± 0.15, and 0.45 ± 0.01 mg quercetin/100 g DM, respectively. The freeze-drying method allowed better retention of flavonoids also in the case of snack C with the addition of treahalose; however, the difference was not so marked.

### 2.4. Antioxidant Activity

**Figure 4:**
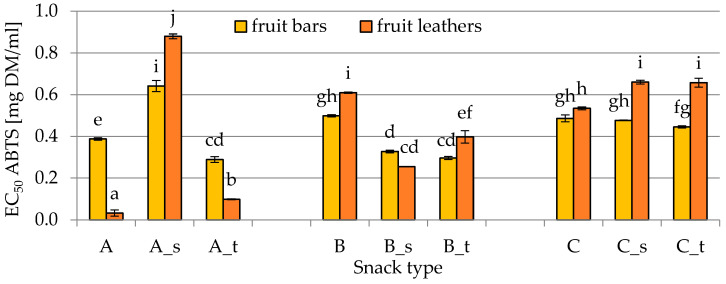
EC_50_ of fruit bars and leathers (A, B, C—different composition; s—sucrose addition; t—trehalose addition) obtained using the ABTS method, as described in Section 4.2.4. Different letters above the columns indicate significant differences (*α* = 0.05) between all considered samples.

Figure 4 presents the antioxidant activity in fruit leathers and bars. Antioxidant activity was express as EC_50_ coefficient, which represents the amount of extract obtained from dried snacks required to scavenge 50% of radicals. Therefore, a low EC_50_ value relates to a high antioxidant activity of the extract. EC_50_ for fruit bars type C with the addition of sugars showed antioxidant activity significantly higher than in fruit leathers. Snack B with the addition of trehalose showed the same trend, while no significant differences were found when sucrose was used. However, in some cases, ambiguous results were observed. Fruit bars type A without the addition of sugars and with trehalose addition had significantly lower antioxidant activity in comparison with fruit leathers. For example, the scavenging activity of the unsweetened fruit bar (type A) was characterized by a value of 0.39 ± 0.01 mg DM/mL, while fruit leather antiradical activity was equal to 0.03 ± 0.02 mg DM/mL. However, an inverse relationship was noted for a snack with the addition of sucrose; that is, fruit bars presented values of 0.64 ± 0.03 mg DM/mL, while fruit leathers showed values of 0.88 ± 0.01 mg DM/mL.

## 3. Discussion

Fruit and vegetables are a good source of bioactive compounds. The lack of these compounds in a daily diet can increase the risk of several diseases such as atherosclerosis, cancers, faster aging of the body, and heart attacks [17]. It is known that bioactive food components are influenced by individual ingredients that play roles as antioxidants such as vitamin C, polyphenols, flavonoids, and so on.

Vitamin C is the most distinctive nutritional parameter of kiwifruit and the amount of this substance is 105–120 mg/100 g in gold kiwifruit, and it is higher than in green kiwifruit, which has vitamin C content in the range of 65–90 mg/100 g [13]. In fact, the vitamin C content is strictly related to the variety of kiwifruit. Cozzolino et al. [18] showed that the amount of vitamin C in ‘Jintao’ and ‘Hayward’ was 76.1 ± 0.91 mg/100 g FW and 47.7 ± 3.14 mg/100 g FW, respectively. Even higher vitamin C content was found in ‘Jintao’ (148.82 ± 0.31 mg/100 g), according to Ma et al. [14]. Therefore, a high amount of vitamin C content in fruit leathers and bars is mainly owing to the high amount of kiwifruit as a basic ingredient (44–56% in the final product). However, other ingredients also used to produce snacks have a significant vitamin C content. Strawberries contain around 40–70 mg/100 g FW of vitamin C [19]. Fennel contains ascorbic acid in the range of 30.45–31.49 mg/100 g FW, depending on the variety [20]. Moreover, lemon juice and spinach show a vitamin C content in the range of 59.3–76.2 mg/L and 75 mg/100 g FW, respectively [21]. Scientific research confirms that, with the increasing temperature and duration of the thermal process, the content of vitamin C in the fruit and vegetables-based product decreases [22,23,24]. In fact, vitamin C is very sensitive to high temperatures; hence, it is often considered as an indicator of the overall quality of the product. It can be stated that, if the used processes did not adversely affect the content of vitamin C, other valuable nutrients have probably also been preserved [25,26]. In the present study, the fruit bars obtained by freeze-drying at a low temperature (40 °C) showed a higher retention of vitamin C content in comparison with fruit leathers obtained by air drying at 70 °C. However, the stability of vitamin C also depends on other factors. Hiwilepo-van Hal et al. [25], studying the effects of heating of three different fruit pulp on the kinetics of vitamin C degradation, found that vitamin C in morula pulp was up to 15 times more stable than in mango or guava pulp. Martínez-Navarrete et al. [27] observed a very high retention of vitamin C (1–1.7 mg of vitamin C/g snack) in freeze-dried snacks based on mandarin juice, when the temperature of 40 °C was used during freeze-drying. In addition, intensive access of air can cause enzymatic oxidation of vitamin C and non-enzymatic oxidation can also occur [26]. The freeze-drying works under reduced pressure and at low temperatures. These conditions have an impact on the quality of products and the freeze-drying is considered the best method of drying. Owing to reduced access to the air during the process, dried fruit and vegetables retain vitamins and bioactive compounds at a high level [28]. Moreover, the texture of freeze-dried products is porous and totally different in comparison with the texture of products obtained by other methods of drying [10]. However, the open structure of freeze-dried materials, owing to easy access to oxygen through the porous structure, may lead to higher levels of degradation of vitamins and bioactive compounds. To avoid the negative effect, proper packaging is required, but some additional treatments may also be used as foaming the puree before drying or addition of hydrocolloids and maltodextrin. Even if there was no significant effect of the addition of sugars on the vitamin C content, it should be taken into consideration that sucrose addition may have an impact on glass transition temperature Tg, which is a key factor influencing the stability of dried food during storage [29].

The obtained fruit leathers and bars were a good source of polyphenols, thanks to the high amount of these compounds, mainly in kiwifruit ranging from 58.45 to 152 mg GAE/100 g FW [14]. In fresh kiwifruit cultivar Jintao, the TPC ranges between 124–145 mg GAE/100 g FW [14,18], while flavonoids, which are the most abundant polyphenols in kiwifruit, show values about 120 mg CTE/100 g FW Ma et al. [14].

In general, the drying process reduces the TPC compared with the fresh samples, as observed by Tylewicz et al. [30] in strawberry samples enriched with bilberry juice-based solution. During drying, the activation of oxidative enzymes (e.g., peroxidase and polyphenol oxidase) could occur, thus causing the degradation of phenolic compounds, which in turn results in decreasing of the nutritional value, loss of sensorial characteristics, browning, and off-flavor development [31].

Wojdyło et al. [32] observed a clear decrease in the content of polyphenols in the cherries dried by hot air, which increased with an increase of the temperature, while better retention of polyphenolic components was found for cherries dried by freeze-drying. This was also observed in our study in snack C without sugar and with the addition of sucrose. Chin et al. [33] analyzed the effect of dried green kiwi slice thickness (0.3 cm and 0.6 cm) and hot air temperature (40–60 °C) on the quality of the obtained products. They found that the use of thinner slices was more beneficial owing to the better maintenance of polyphenols (31–38% lower polyphenol losses compared with thicker slices). Kiwifruit slices of 0.3 cm thickness dried at 40, 50, and 60 °C contained 842, 751.31, and 958.70 mg gallic acid/100 g DM, respectively. Such good behavior of phenolic components at 60 °C was explained by the availability of their precursors resulting from non-enzymatic interconversion and the shorter duration of the drying process, which causes less exposure to thermal and oxidative degradation [33]. However, high TPC might be linked with the research method, where Folin-Ciocalteu reagent is used [34].

Concerning the flavonoid content, a smaller values gap between freeze-dried and convective dried snacks C could be linked to the difference in its formula—unlike A and B snacks, it did not contain lemon juice, nor lemon peel. Lemons, in fact, are particularly rich in flavonoid compounds [35]. Among all the snacks, the unsweetened freeze-dried one with fennel and lemon (juice and peel) was marked by the highest content of flavonoids. The changes in flavonoid content during processing are very complex and depend on various factors. Some researchers observed its decline as a result of heat treatment (caused by its degradation), as observed in unsweetened snacks A and B and in snack A with the addition of trehalose (A_t). Increased content of flavonoids in fruit bars with addition of trehalose may indicate that trehalose preserved lipid bilayers during dehydration, and thus protected some bioactive compounds [36]. Moreover, notable differences in flavonoid content among analyzed kiwifruit snacks could occur for the unstable behavior of flavonoids during storage.

Antioxidant capacity determines the potential of samples to reduce pro-oxidative and reactive substances such as free radicals [37]. In many studies, a clear positive correlation has been observed between the content of phenolic compounds and the antioxidant capacity of fruit and vegetables [28]. The antioxidant activity is influenced by many factors like plant variety, growing season, ripening, harvest time, storage time, and processing. Especially during the thermal food processing, the bioactive food components are significantly reduced [21,24,35]. Generally, fruit bars obtained in our study were characterized by a similar or higher content of antioxidant activity in comparison with the fruit leathers, apart from two samples of formulation A, which showed an opposite trend. EC_50_ for fruit bars type C with the addition of sugars was significantly higher than that of fruit leather of the same formulation. This indicates that the freeze-drying process is more favorable in terms of maintaining antioxidant capacity. This was also observed by Materska [28], who found that freeze-dried fruits attain a little less antiradical activity in comparison with fresh material. However, in some cases, ambiguous results were observed. A snack variant A unsweetened and with the addition of trehalose had significantly lower antioxidant activity, while an inverse relationship was noted for a snack with the addition of sucrose. Such a behavior may be linked to the extraction process, where not only antioxidants, but also compounds with antagonistic activity may be transferred from the sample to the solvent, which may distort the final result [38]. Moreover, Orikasa et al. [26] showed that hot air drying with the temperatures in the range of 50–70 °C did not cause significant changes in antioxidant activity. The results obtained in the present study, which indicates non statistical differences between fruit bars and leathers (samples B_s and C), as well as those indicating the greater antioxidant capacity of fruit leathers (samples A and A_t), may also be associated with the phenomenon observed by Vega-Gálvez et al. [39]. Researchers analyzed Granny Smith apple slices dried at three temperatures, 40, 60, and 80 °C, at different airflow rates, that is, 0.5, 1.0, and 1.5 m/s. The antioxidant capacity of samples dried at 40 and 80 °C did not differ significantly, despite the differences in the content of phenolic compounds in these samples, which, according to researchers, was associated with progressive Maillard reactions and parallel transformations that led to the accumulation of antioxidants in the apple slices. Moreover, the higher or comparable concentration of bioactive compounds in the hot-air dried leathers than in the freeze-dried formulations might be related to the processes that occur during preparing and drying snacks. When all ingredients were mixed, the integrity of the cells was ruptured and the enzymatic oxidation took place [40]. The procedures for preparing the leathers and freeze-dried materials were different and took different amounts of time. For freeze-drying, the sample needs to be frozen before drying, and in the samples in the freezing state (in our study, they remained frozen for 48 h), oxidation does not stop, but only slows down [41]. Moreover the freeze-drying process, even at a low temperature and under reduced pressure, lasted around 24 h, while hot-air dried leathers were obtained after 6 h. Finally, the structure of the freeze-dried material is much more porous [10] than leathers, which means that the oxidation occurs much more easily and the higher reduction of bioactive compounds [29] and antioxidant ability might be observed. Moreover, it is worth emphasizing that polyphenols are known as a major group of strong antioxidants [17,40] that highly affect antioxidant activity. However, the Folin-Ciocalteu reagent might detect not only polyphenols, but also other compounds that easily donate electron (as reducing sugars, vitamin C, amino acids, proteins, carbohydrates, and aromatic amines), which causes an overestimation of the final value [34].

## 4. Materials and Methods

### 4.1. Snack Preparation

#### 4.1.1. Materials and Puree Preparation

Yellow kiwifruits (*Actinidia chinensis* ‘Jintao’) with caliber lower than 65 g were used as the basis for the preparation of fruit snacks. The kiwifruits with similar ripening degree were provided by Jingold Spa (Cesena, Italy). Other fruit and vegetables used for puree preparations were fennel, spinach leaves, strawberries, and organic lemons (with peel suitable for consumption). All the ingredients were purchased on the local market (Apofruit, Cesena, Italy). Until the research, the fruit and vegetables were stored in refrigerated conditions at 4 ± 1 °C. Pectin derived from citrus peels was used as a gelling agent (Sigma-Aldrich, Steinheim, Germany) and refined beet sugar (Eridania Italia SPA Bologna, Italy) and trehalose (EXACTA + OPTECH Labcenter S.p.A., San Prospero, Italy) were used as sweeteners. All the chemicals, including aluminum chloride, Folin-Ciocalteu reagent, ABTS, and standards, were used from the same source (one package) for analysis of all obtained samples. Furthermore, the analysis was conducted in a short timeframe. Furthermore, the aluminum chloride (Sigma-Aldrich, Steinheim, Germany) was fresh and stored under anhydrous conditions (under CaCl_2_ in room temperature) and the solution was prepared before the analysis.

**Figure 5:**
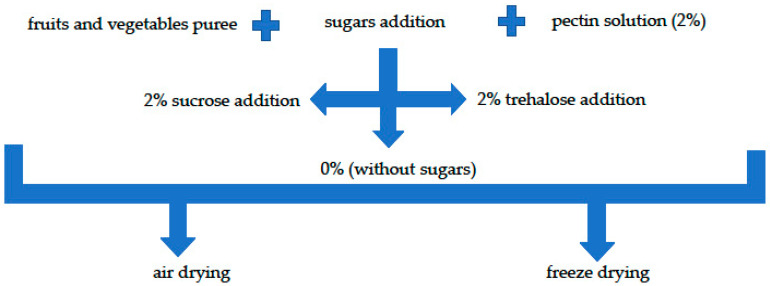
Snacks’ preparation procedure.

In preliminary studies, different technological trials (data not shown) were carried out to create snacks with the desired characteristics. Fruit and vegetables were washed and peeled, and inedible parts were removed before further processing. The fruit and vegetables were ground in a blender (Electrolux ESB9300, Milan, Italy) according to the recipes shown in Table 1 to achieve a homogeneous pulp. Then, the sweeteners (sucrose or trehalose) were added to puree to the final concentration of 2% (*w*/*w*). The puree without sweetener addition was used as the control sample. A 2% solution of pectin, previously prepared by dissolving pectin in hot water and cooled to 70 °C, was added in a ratio of 4:1 (80% of fruit and vegetables and 20% pectin solution at 2% (*w*/*v*)) to all purees and mixed. The snack preparation process is shown in Figure 5.

#### 4.1.2. Fruit Leathers Preparation by Air Dying

Fifteen grams of each formula was spread to a 200 × 300 mm layer, on laminated paper sheets, and then placed on holed metal plates to ensure air circulation. The metal plates were placed in the hot air cabinet dryer (POL-EKO-APRATURA SP.J., Wodzislaw Slaski, Poland), which was previously set at 70 °C with air velocity of 2 m/s and an air renewal fee of 50%. The drying was performed for 6 h, and after drying, the fruit leathers were removed from the laminated paper and cut into strips of about 20 × 50 mm. Until the analysis, the fruit leathers were stored in hermetic under vacuum packaging. Three independent drying cycles were carried out.

#### 4.1.3. Fruit Bars Preparation by Freeze-Drying

Each formula (15 g) was placed into plastic molds (25 × 20 × 20 mm) and then frozen at −40 °C and kept in the freezer for 48 h. The frozen samples were removed from the molds and dried in a freeze-dryer (Lio2000, CinquePascal S.r.l., Milan, Italy) at 40 °C and 25.12 Pa. Until the analysis, the fruit bars were stored in hermetic packaging. Three independent drying cycles were carried out.

### 4.2. Chemical Analysis

#### 4.2.1. Vitamin C Content

The analysis was performed using liquid chromatography with a photodiode detector (WATERS Acquity UPLC H-Class, Milford MA) [42]. To 50 mg of ground sample, 20 mL of cold extraction solution (3% metaphosphoric acid, 8% acetic acid, 1 mM EDTA) was added; the mixture was stirred for 10 min and centrifuged for 6 min at 4 °C and 6000 rpm. The supernatant was filtered (0.22 µm polytetrafluoroethylene—PTFE syringe filter, Sigma-Aldrich, Germany), diluted twice with eluent, and injected in the amount of 10 µL. All operations were conducted with limited access to light. Samples were extracted directly prior to analysis. WATERS Acquity HSS T3 column (100 × 2.1 mm, 1.8 μm particle size) with pre-column BEH C18 (2.1 × 5 mm, 1.7 µm particle size) was used for separation. The mobile phase (Milli-Q water with 0,1% (*v*/*v*) formic acid) flow was 0.25 mL/min, the autosampler temperature and the samples were kept at 4 °C, and the column temperature was 25 °C. Absorbance was monitored at 245 nm. Vitamin C content was calculated from a calibration curve for the L (-) ascorbic acid standard (Sigma-Aldrich, Steinheim, Germany) (0.005–0.100 mg/mL) and the results were expressed as mg/100 g dry matter (DM). The analysis was performed in triplicate.

#### 4.2.2. Total Polyphenol Content (TPC)

The total polyphenol content of the samples was determined by the Folin-Ciocalteu method [24]. The samples were ground (A11 basic, IKA- Labortechnik, Germany) and 0.3 g was mixed with 20 mL of 80% ethanol solution. The mixture was homogenized (Ultra-Turrax T-10, IKA- Labortechnik, Staufen, Germany), heated on a hot plate under cover until boiling, and then filtered through a filter paper into a flask and made up to 50 mL by ethanol solution at 80%. The 0.18 mL of as prepared extract was mixed with 4.92 mL of water and stirred. Then, the 0.3 mL of Folin-Ciocalteu reagent (POCH, Gliwice, Poland) was added and mixed again. After 3 min, 0.6 mL of a sodium carbonate solution was added and stirred. The mixture was left at room temperature in the dark. After 30 min of incubation, absorbance at 750 nm was measured against a blank (ethanol was used instead of the extract). The TPC was calculated based on the calibration curve prepared for the gallic acid standard (Sigma-Aldrich, Steinheim, Germany) in the range of 1–5 mg/mL. The analysis was performed in triplicate and the results were expressed as mg of gallic acid (GAE) in 100 g of dry matter of the sample.

#### 4.2.3. Flavonoid Content

The total flavonoid content in the samples was measured according to a spectrophotometric method based on the colored reaction of flavonoid compounds with aluminum chloride [42]. Here, 2 mL of ethanolic extract (the preparation of the extract is described in Section 4.2.1) was mixed with 2 mL of 2% aluminum chloride solution (in 80% ethanol). After 10 min of incubation at 25 °C, in the absence of light, the absorbance at 430 nm was measured using a UV/VIS spectrophotometer (Thermo Spectronic Helios Gamma, Thermo Fischer Scientific, Waltham, MA, USA). The absorbance of colored extract solutions was corrected by measuring a mixture of 2 mL ethanol with 2 mL of 80% ethanol solution. The flavonoid concentration was calculated from a calibration curve in the range of 0.5–200 µg/mL of quercetin standard (Sigma-Aldrich, Steinheim, Germany) and the result was expressed as mg of equivalent quercetin in 100 g of dry matter. The analysis was performed in triplicate.

#### 4.2.4. Antioxidant Activity

The antioxidant activity of the dried snacks was determined by the ABTS method according to Nowacka et al. [43]. The free radical solution was prepared by dissolving 0.0384 g of 2,2-azino-bis(3-ethylbenzothiazoline-6-sulfonate)—ABTS (Sigma-Aldrich, Steinheim, Germany) and 0.0066 g of potassium persulfate (Sigma-Aldrich, Steinheim, Germany) in 10 mL of distilled water and left the solution in the fridge for 16 h. The working solution was prepared immediately prior to the analysis by dilution with an 80% ethyl alcohol solution of the radical solution; its absorbance at 734 nm should be in the range of 0.680–0.720. The extract (20, 40, 60, 80 µL) and 2 mL of ABTS solution were added to four glass tubes, mixed, and incubated at room temperature in the dark for 6 min. The absorbance was measured at 734 nm with a spectrophotometer (Spectronic 200; Thermo Fisher Scientific Inc., Waltham, MA, USA), using 80% ethyl alcohol as the blank sample. Antioxidant activity (decrease in absorbance expressed as scavenging effect) was determined by calculating according to the following equation:

Scavenging Effect [%] = (Abs_ABTS_ − Abs_Extract_) × 100/Abs_ABTS_ (1)

where Abs_ABTS_ and Abs_Extract_ are the absorbance at 734 nm of ABTS radical solution for blank and sample when the extract was added, respectively.

EC_50_ coefficient was determined based on the dependence of scavenging effect versus the concentration of the sample in the extract. The curve was plotted and the concentration of extract from dried material required to reduce 50% of ABTS radicals (EC_50_ ABTS) was calculated [24]. The measurements were repeated twice for each independent extract.

### 4.3. Statistical Analysis

The results were analyzed with a one-way analysis of variance (ANOVA) using Statistica 13 software (TIBCO Software, CA, USA). Significant differences among the samples were verify using the Tukey test (*α* = 0.05%).

## 5. Conclusions

Fruit bars showed higher total phenolic compounds, vitamin C, and flavonoids content. However, the antioxidant activity did not always follow this trend, showing in some cases (samples A and A_t) higher values of this parameter in fruit leathers. The amount of bioactive compounds depended on the formulation used for the preparation of snacks. The effect of the sugar addition instead seems to be strictly related to the mix used and specific bioactive compound investigated.

Both methods of preparation resulted in the production of attractive snack products that fit into the current trends on the snack market and allowed to reduce the waste of kiwifruits by using yellow kiwifruits, which do not meet the retail criteria. However, in terms of the preservation of bioactive ingredients, such as vitamin C and flavonoids, the use of the freeze-drying process, which led to the development of fruit bars, was more favorable than convective drying used to obtain fruit leathers.

The obtained results could be useful to design novel snacks with a high nutritional value. However, further studies are necessary to better understand the behavior of different specific bioactive compounds as well as their availability during digestion by in vitro and in vivo assays.

## Figures and Tables

**Table 1:** Composition of snacks based on yellow kiwifruit, containing 80% fruit and vegetable puree and 20% pectin solution.

Snack Symbol	Fruit and Vegetable Ingredients (%)	Sugar Addition	Addition of 2% Pectin Solution	Abbre-Viation
A	44% kiwifruit	0	20%	A
	21.6% strawberry	sucrose		A_s
	12% fennel	trehalose		A_t
	2.4% lemon juice			
B	52% kiwifruit	0	20%	B
	24% fennel	sucrose		B_s
	3.88% lemon juice	trehalose		B_t
	0.12% lemon peel			
C	56% kiwifruit	0	20%	C
	16% fennel	sucrose		C_s
	8% spinach	trehalose		C_t
